# Long-term outcome after colic surgery: retrospective study of 106 horses in the USA (2014–2021)

**DOI:** 10.3389/fvets.2023.1235198

**Published:** 2023-10-04

**Authors:** Lisbeth B. Matthews, Macarena Sanz, Debra C. Sellon

**Affiliations:** Department of Veterinary Clinical Sciences, College of Veterinary Medicine, Washington State University, Pullman, WA, United States

**Keywords:** colic, surgery, survival, return to work, long term outcome, exploratory celiotomy, complications, satisfaction

## Abstract

**Objective:**

To determine long-term survival rate, defined as survival to 1 year after discharge from the hospital, and rate of return to prior athletic, breeding, or other function in horses undergoing colic surgery at the first presentation at one referral hospital in the Pacific northwest region of the United States.

**Procedures:**

Records were reviewed for all horses that underwent colic surgery between October 2014 and October 2021. Owners of horses that survived to discharge were contacted to obtain follow-up information via internet-based questionnaire or telephone interview. The percentage of horses that survived to 1 year after discharge, rates of return to function, and complications occurring after hospital discharge were determined. The possible association of patient signalment, lesion type, and surgical procedures with long-term survival was examined with Chi-square and Fisher’s exact tests. Overall satisfaction was indicated on a scale of 1 (unsatisfied) to 5 (very satisfied).

**Results:**

Of 185 horses that underwent surgical intervention for colic and met the inclusion criteria, 134 horses recovered from anesthesia, with 106 of these recovered horses (79.1%) surviving to discharge. Of the 71 horses for which follow-up information was obtained, 61 horses survived to 1 year after discharge (long-term survival rate of 85.9%). There were no demographic, lesion type, or surgical procedure variables significantly associated with long-term survival. Prior to surgery, 59/71 horses (83.1%) were engaged in some type of athletic activity. After surgery, 44/61 horses (72.1%) were reported to be athletically active. Only one horse was reported to be retired from athletic activity as a direct result of a complication from surgery. Overall satisfaction with the decision to proceed with colic surgery was rated by all respondents as 4 or 5.

**Conclusions and clinical relevance:**

The long-term survival for this sample of horses was similar to previously published reports of long-term survival after colic surgery. Horses that survived to hospital discharge were highly likely to remain alive and be athletically active 1 year later. There were no factors related to the signalment of the horse, the specific cause of colic, or the surgical procedures performed that were significantly associated with likelihood of survival.

## 1. Introduction

Emergency colic cases make up 55–61% of all equine emergencies in referral practice, with 23–45% of these horses requiring colic surgery (1–3). Colic surgery is an emotional, financial, and time-consuming investment for horse owners. In addition to substantial costs directly associated with surgery and critical care, there are costs associated with stall rest, loss of training, and loss of competition or breeding time. It is important that owners make informed decisions regarding medical care for their horses. It is estimated that 9% of colic cases seen by primary care veterinarians require additional hospital management (3). Factors thought to be important in owners’ decisions to consent to colic surgery for their horse include prognosis for survival, personal opinions or experiences, financial considerations, ability to travel, age of the horse, insurance status, and prognosis for continued performance (4, 5).

Recent studies report a high owner satisfaction rate of 76–98.5% after colic surgery (4, 6, 7). The only factor found to affect owner satisfaction was return to work while post-operative complications appeared to play no significant role (5). In horses that have undergone colic surgery and are discharged from the hospital, repeat colic episodes are the most frequently described complication, with 20–50% of horses experiencing one or more episodes of abdominal pain. This is estimated to be a 1.7–7.6 times higher incidence than in horses with no prior history of colic surgery (7–10). Incisional site infections and abdominal wall hernias are also frequently reported complications, at 11–42% and 6–17%, respectively, (7, 8, 10, 11). An estimated 70–95% of horses return to competition work by 1 year after surgery (6, 7, 12, 13). Presence of an abdominal wall hernia is associated with a 7–14% decreased probability of returning to athletic use. Of all horses that returned to previous athletic activities, 70–91% were working at the same or a higher level as compared to pre-surgical performance (6, 7, 14, 15).

Short- and long-term survival outcome after surgery has been defined in several ways (7, 8, 10, 12, 16–22). Long-term survival after colic surgery, commonly defined as any period of time after hospital discharge up to 1 year after surgery, is reported to be 66–91% with large regional and population variations (7, 8, 10, 17, 18, 21, 23). In the past 10 years, reports of long-term survival and performance after colic surgery have originated from European countries with minimal information from United States (US) horse populations. Population characteristics, cultural norms, and geographical considerations affect types of colic, decision-making by owners, and details of case management that can impact survival and long-term outcome for horses undergoing colic surgery (12, 24–28). The US equine population varies from that of Europe, and it is unclear whether data can be accurately extrapolated between these populations (29, 30). Thus, it is important to investigate and to report national and regional colic survival outcomes and risk factors for specific populations (20, 22). The objective of this study was to describe the survival of horses following colic surgery at a referral hospital within the Pacific Northwest region of the US.

## 2. Materials and methods

### 2.1. Data collection

Medical records were retrospectively reviewed for all horses and ponies aged ≥1 year undergoing abdominal surgery at the Washington State University Veterinary Teaching Hospital via either ventral midline celiotomy or standing flank laparotomy between October 2014 and October 2021. Horses that underwent more than one abdominal surgery intervention during a single period of hospitalization were included in the analysis once with follow-up data collected after discharge from the hospital as for all other horses. Horses that had multiple surgeries across different hospitalization events were included only once in the analysis with follow-up data collected after discharge from first surgery. Colic episodes that resulted in subsequent hospitalizations and surgical interventions, after initial discharge, were considered complications of the initial surgery. Short-term survival was defined as survival to discharge from the hospital. Long-term survival was defined as survival for a minimum of 1 year after the date of discharge.

Colic was defined as acute or recurrent signs of abdominal discomfort. Donkeys and mules were excluded from analysis. Horses euthanised during surgery for financial reasons or because of poor prognosis or that died prior to full recovery from anesthesia were excluded from the final analysis. Data extracted from each medical record included age, breed, sex, surgery date, surgical diagnosis, surgical procedures, complications during or after surgery, and days to discharge from hospital after surgery. Surgical diagnoses were categorized as primary large intestinal disease (including cecal disorders) or primary small intestinal disease and as either strangulating, non-strangulating, or other type of lesion (e.g., colitis, peritonitis, enteritis). Surgical procedures were categorized as exploratory only, enterotomy, or intestinal resection. Breed information was curated into categories designated as stock-type horses (Quarter Horse and American Paint Horse), other light horse breeds, Warmblood breeds, and all other breeds including American Miniature Horse and draft horse breeds. Distance travelled to hospital was established using the zip code provided in the medical record and distance in miles was calculated from Google maps.

Follow-up data was collected from horse owners using a questionnaire that could be completed via telephone interview or on-line data entry. The owners of horses that were discharged were contacted via phone and email to request participation in the long-term outcome study questionnaire.

### 2.2. Questionnaire

A questionnaire for horse owners was designed on a commercial internet survey site (Qualtrics, Provo, UT) and included 27 items separated into five sections: introduction, medical record and demographic information, post-surgical complications, return to physical activity, and owner satisfaction. Owners with emails on file were sent a link to the on-line questionnaire with information describing the study. Owners who did not complete the questionnaire or did not have an email address in the medical record were contacted via telephone and offered the option of providing information via a structured interview. For these owners, questions were read aloud, and an investigator entered the information directly into the questionnaire. If preferred, the link to the questionnaire was resent via email. Questions related to events that occurred between the date of discharge from the hospital until either the date of the horse’s death or the end of the study period. The questionnaire was initiated on March 1, 2023, and remained open for 2 months. The full text questionnaire is available as Supplementary Item 1.

Respondents were asked to specify if their horse was alive 1 year after discharge from hospital. If the horse was not alive at the one-year time point, respondents were asked to provide the cause and approximate date of death. Respondents were asked to describe athletic and life function prior to surgery and after discharge from the hospital and whether their horse returned to original athletic or life function at any point after discharge.

Respondents were asked whether their horse experienced post-surgical complications after discharge from the hospital. Specific questions were asked related to the number and severity of colic episodes. The other complications described by respondents were curated into categories including abdominal wall hernia, diarrhea, fever, laminitis, peritonitis, surgical site infection, or other complication based on the description provided by the owner. Each horse was then categorized as to whether they experienced one or more episodes of colic after discharge and whether they experienced any other type of complication after discharge.

The final section of the questionnaire sought to determine the overall level of satisfaction each respondent had with their decision to proceed with colic surgery on their horse. Overall satisfaction was rated using a graphic slider question with a 5-point scale in which satisfaction ranked from poor (angry cartoon face) to highly satisfied (large cartoon smile). Each response was classified on a visual analog scale as very unsatisfied (1), unsatisfied (2), neutral (3), satisfied (4), or very satisfied (5). The final question asked whether each respondent would proceed with colic surgery for another horse, would elect euthanasia, or was unsure of that decision.

### 2.3. Data analysis

Statistical analyses were performed using commercial statistical software (SigmaStat 4.0, Systat Software, Inpixon, Palo Alto; GraphPad, Dotmatics, Boston). Responses were summarised in tabular form. Normality of data was assessed with Shapiro–Wilk test. Descriptive statistics were determined as appropriate including mean and standard deviation or median with an interquartile range (IQR) that represented the 25^th^ and 75^th^ quartiles. Confidence intervals for proportions were calculated using the modified Wald technique. The primary outcome variable for analysis was status at 1 year after discharge from the hospital (alive or dead). For this dependent variable, categorical variables were compared in univariate analysis by Chi-square analysis or Fisher’s Exact test.

## 3. Results

### 3.1. Study population

**Figure 1:**
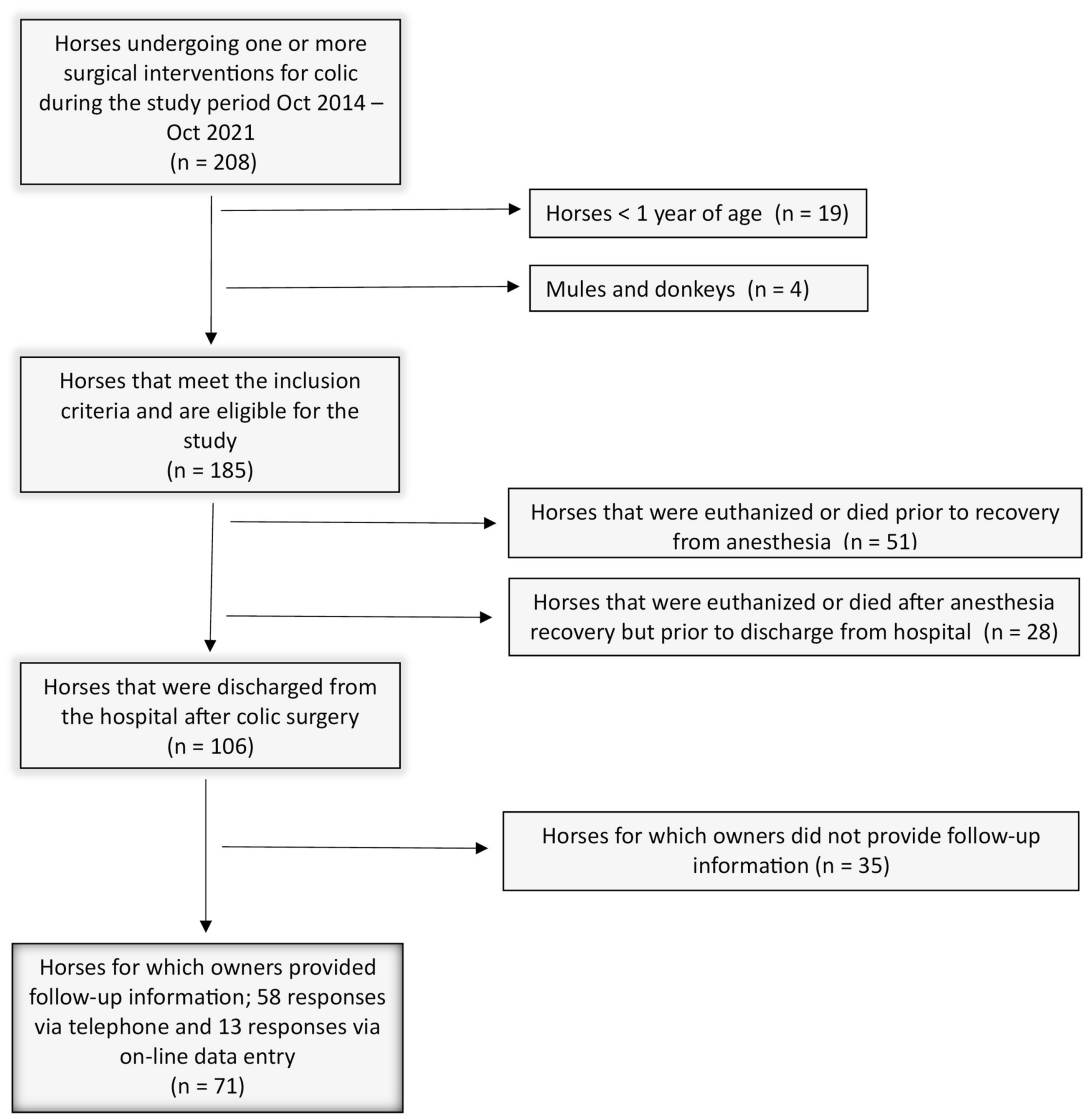
Flow chart of the inclusion criteria to determine the study population.

A total of 208 horses underwent exploratory celiotomy (*n* = 207) or laparoscopy (*n* = 1) for treatment or diagnosis of colic during the study period (October 2014 – October 2021). Because there was only one horse for which surgery was performed by laparoscopy, data from all horses were analyzed together regardless of the type of surgery. Horses <1 year of age (*n* = 19) and all mules and donkeys (*n* = 4) were excluded from analysis. Data from 185 horses was included in the final data set (Figure 1). Of these 185 horses, 13 horses had repeat colic surgeries at WSU, with one horse undergoing two additional surgeries in the same hospitalisation period. Each of these 13 horses was included in the analysis once based on the date and events of the first surgery. Follow-up information was available from 71 owners.

Of the 185 horses that met the inclusion criteria, median age was 12 years (IQR = 8–17 years, range = 1–29 years). Quarter Horses were the most prevalent breed (*n* = 75, 40.5%) followed by American Paint Horses (*n* = 19, 10.3%). The category “Other” included 19 horses (10.3%) and consisted of Friesian (*n* = 8), Paso Fino (*n* = 1), Pony of the Americas (*n* = 1), Shagayi (*n* = 1), Norwegian Fjord (*n* = 2), unspecified (*n* = 1), and mixed breeds (*n* = 4). There were 80 mares (43.2%), 86 geldings (46.5%), and 19 stallions (10.3%). The median distance travelled to the hospital at the time of colic was 121 miles (IQR = 97–160.8 miles, range = 5.8–809 miles).

### 3.2. Surgical lesions and procedures

Of the 185 horses included in the analysis, 134 horses (72.4%) recovered from anesthesia and surgery; 51 horses (27.6%) were euthanized during surgery due to financial reasons or poor prognosis for survival or died prior to recovery. Of the 134 horses that recovered from anesthesia, 106 horses (79.1%) survived to discharge from the hospital.

Large intestinal lesions were most common (*n* = 102/185, 55.1%) and included large colon impactions (*n* = 38/185, 20.5%), large colon displacements (*n* = 38/185, 20.5%) large colon volvulus (*n* = 21/185, 11.4%), and lesions affecting the cecum (*n* = 5/185, 2.7%). Small intestinal lesions (*n* = 49/185, 26.5%) and small colon lesions (*n* = 13, 7.0%) were less common. Other types of lesions were identified in 21 horses (11.4%) and consisted of inflammatory lesions (*n* = 11/185, 5.9%), neoplasia (*n* = 1, 0.5%), ruptured viscus (*n* = 5, 2.7%), and gastric impaction (*n* = 1, 0.5%) For 3 horses (1.6%), a definitive diagnosis was not obtained. Of the 185 horses undergoing colic surgery, strangulating lesions were identified in 59 horses (31.9%), a large colon enterotomy was performed in 85 horses (45.9%), and intestinal resection and anastomosis was performed in 22 horses (11.9%).

### 3.3. Long-term survival

**Figure 2:**
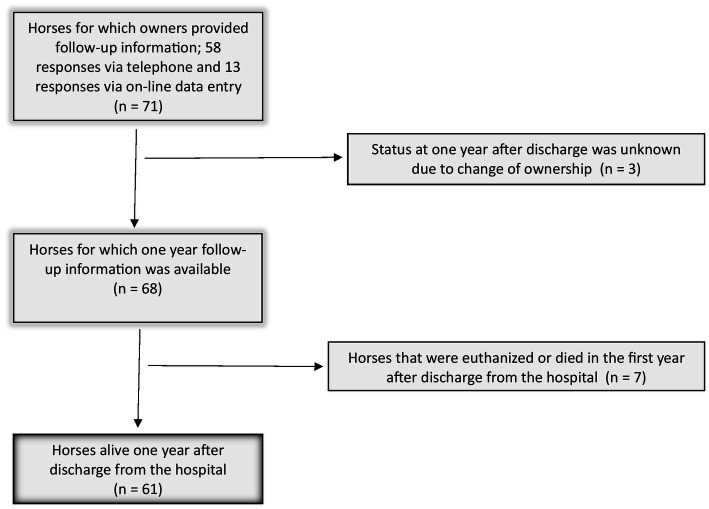
Flow chart of the horses who were alive 1 year after discharge for which follow-up information was available.

Invitations to complete the follow-up questionnaire were sent to owners of 106 horses that were discharged from the hospital after surgery. Responses were received from 71 owners (67.0%). Status at 1 year after discharge from the hospital was unknown for 3/71 horses (4.2%, Figure 2) due to change of ownership. Of the 68 horses for which one-year follow-up information was obtained, most horses were alive at 1 year after discharge (*n* = 61/68, 89.7%). Seven horses (9.9%) were either euthanised or died within the first 12 months after discharge from the hospital. For 4 of these 7 horses, owners reported euthanasia or death was possibly related to complications with the colic surgery (57.1%). At the conclusion of the study, 56 of 68 horses discharged from the hospital were confirmed to remain alive (82.4%) for follow-up periods ranging from 2–9 years post-surgery. Information related to timing and cause of death of the 12 horses which were no longer alive at the time of the follow-up questionnaire is shown in Table 1. Demographic and surgical variables that were assessed were not significantly associated with survival outcome of 61 horses that survived to 1 year (Table 2). Horses that were alive at 1 year travelled a median distance of 134 miles (IQR = 100–161 miles) from their premises to the hospital and horses that were not alive at 1 year travelled a median distance of 143 miles (IQR = 123–184 miles) (*p* = 0.4).

**Table 1:** Type of colic, surgical procedures, time of death, and cause of death for 12 horses that were no longer alive at time of owner contact.

Horse	Cause of colic	Surgical procedure	Time of death in relation to initial hospital discharge	Cause of death
1	Large colon impaction	Enterotomy	3 to 4 months	Colic, euthanized on the farm
2	Large colon impaction	Enterotomy	< 1 year	Laminitis
3	Large colon impaction	Enterotomy	< 1 year	Seizures of undetermined origin
4	Small intestinal strangulating obstruction	Resection and anastomosis	4 to 5 days	Colic, euthanized during surgery
5	Large colon volvulus	Enterotomy	2 to 3 months	Unresponsive fever
6	Large colon displacement	Large colon displacement	< 1 year	Osteoarthritis
7	Large colon impaction	Enterotomy	2 months	Undetermined
8	Small intestinal strangulating obstruction	Resection and anastomosis	> 1 year	Colic, euthanized during surgery
9	Large colon displacement	Exploratory only	2 years	Renal failure
10	Large colon impaction	Enterotomy	5 years	Undetermined
11	Large colon impaction	Enterotomy	3 years	Colic
12	Large colon volvulus	Exploratory only	5 years	Colic

**Table 2:** Results of Chi-square analysis and 95% confidence intervals (CI) of proportions for demographic and surgical description variables for 68 horses for which 1-year post-surgery survival data was available.

Variable	Alive at 1 Year			Not Alive at 1 Year			*p*-Value
	Number	%	95% CI	Number	%	95% CI	
≤ 5 years of age	9	75.0%	46–92%	3	25.0%	8–54%	0.1
6–15 years of age	39	95.1%	83–100%	2	4.9%	0.5–17%	
≥ 16 years of age	13	86.7%	61–98%	2	13.3%	2–39%	
Quarter Horse or American Paint Horse	28	93.3%	78–99%	2	6.7%	0.8–22%	0.2
Other light horse breed	16	94.1%	71–100%	1	13.3%	0.01–29%	
Warmblood breed	12	80.0%	54–94%	3	6.7%	6–46%	
Other breed	5	83.3%	42–99%	1	16.7%	1–58%	
Warmblood breed	12	80.0%	54–94%	3	20.0%	6–46%	0.2
All other breeds	49	92.5%	82–98%	4	7.5%	2–18%	
Gelding	25	89.3%	72–97%	3	10.7%	3–28%	1.0
Mare	27	90.0%	74–97%	3	10.0%	3–26%	
Stallion	9	90.0%	57–100%	1	10.0%	<0.001–43%	
Lesion of large colon, small colon, or cecum	49	89.1%	78–95%	6	10.9%	5–22%	0.7
Lesion of small intestine	7	87.5%	51–100%	1	12.5%	0.1–49%	
Other or undetermined lesion	5	100.0%	51–100%	0	0.0%	0–49%	
Strangulating lesion	12	92.3%	65–100%	1	7.7%	<0.001–35%	1.0
Nonstrangulating lesion	44	88.0%	76–95%	6	12.0%	5–24%	
Resection and anastomosis performed	3	75.0%	29–97%	1	25.0%	3–71%	0.4
No resection and anastomosis performed	58	90.6%	81–96%	6	9.4%	4–19%	
Enterotomy or resection performed	40	87.0%	74–94%	6	13.0%	6–26%	0.4
No enterotomy or resection performed	21	95.5%	76–100%	1	4.5%	0.001–24%	

Of the 71 horses discharged from the hospital for which follow-up information was obtained, 36 had some type of complication, including colic, incisional site infection, abdominal wall hernia, laminitis, peritonitis or other, after discharge (50.7%). Respondents were specifically asked how many episodes of colic their horse had experienced since discharge from the hospital. One or more episodes of abdominal pain were reported in 21 of 70 horses (30.0%, Table 3). Two horses (2.8%) required a second colic surgery after discharge. Excluding colic, 47 of 71 horses (66.2%) had no known complications after discharge and 24 horses had at least one post-surgical complication other than colic (Table 4). Laminitis was reported in 4.2% of horses (*n* = 3/71) with 2 of these horses requiring euthanasia within the first year after discharge.

**Table 3:** Distribution of the number of post-operative colic episodes across the 71 horses for which follow-up information was available after discharge.

Number of episodes	Number of horses	% of Horses (95% CI)
0	49	69.0% (57–79%)
1	11	15.5% (9–26%)
2	4	5.6% (2–14%)
3	2	2.8% (0.2–10%)
4+	4	5.6% (2–14%)
Not reported	1	1.4% (< 0.001–8%)

CI, confidence interval.

**Table 4:** Distribution of post-operative complications across the 71 horses for which follow-up information was available after discharge.

Complications	Number of horses	% of Horses (95% CI)
Abdominal wall hernia	4	5.8% (2–14%)
Diarrhea	1	1.4% (<0.001–8%)
Fever	2	2.9% (0.2–10%0)
Laminitis	3	4.3% (1–12%0)
Peritonitis	1	1.4% (<0.001–8%)
Surgical site infection	6	8.7% (4–18%)
Other	5	7.2% (3–16%)
None	47	68.1% (55–76%)

CI, confidence interval.

Of the 71 horses with follow-up information, pre-surgical activities included some type of athletic function for 59 horses (83.1%). Prior to surgery these horses were engaged in English-style competition (*n* = 18/71, 25.4%), Western-style competition (*n* = 10/71, 14.1%), or non-competitive pleasure riding (*n* = 31/71, 43.7%). The remaining horses were described as pasture horses with no athletic or breeding function (*n* = 4/71, 5.6%) or breeding animals (*n* = 8/71, 11.3%).

There were 44 of 61 horses (72.1%) reported to be engaged in some athletic activity after surgery. These reported activities included 15 horses in English-style competition (26.2%), 9 in Western-style competition (18.0%), 20 in non-competitive pleasure riding (34.4%). Of the remaining 17 horses, 8 returned to breeding functions (13.1%) and 9 were pasture horses (14.8%). Of the 9 horses reported to be pasture horses, 4 were pasture horses prior to surgery. Only one respondent stated that a complication secondary to surgery was the primary reason to retire the horse from physical activities. Of the 68 horses for which 1 year post discharge follow-up information was obtained, 52 horses returned to previous or intended function (76.4%), including breeding or athletic activity.

### 3.4. Owner satisfaction

Respondents (*n* = 71) were asked to rate their level of satisfaction after surgery using a graphical slider interface based on cartoon facial expressions. Results were interpreted and analyzed using a scale of 1–5 (1 = very unsatisfied, 2 = unsatisfied, 3 = neutral, 4 = satisfied, and 5 = very satisfied). Of 63 responses, all respondents recorded a satisfaction level of 4 or 5 (100.0%) with 58 respondents (92.1%) providing a rating of 5 and 5 respondents providing a rating of 4 (7.9%). When asked whether they would choose surgery for another horse in a similar situation in the future, 52 of 69 respondents said yes (75.4%), with 2 choosing no for another surgical intervention (2.9%) and 15 respondents were unsure at this time, with the decision depending on financial and emotional connection (21.7%). Of the 2 respondents who responded “no” when asked about a future colic surgery, both horses were alive at the time of follow-up (more than 1 year following discharge) and both horses had returned to some level of function. Each horse developed post-operative complications of either peritonitis (*n* = 1) or surgical site infection (*n* = 1).

## 4. Discussion

This retrospective study evaluated the long-term outcome, defined as survival to 1 year after hospital discharge, of horses undergoing surgical intervention for colic at the Washington State University Veterinary Teaching Hospital. More than half of the horses experienced some type of post-surgical complication after discharge from the hospital, the most common of which was one or more repeat episodes of abdominal pain. Despite this subjectively high prevalence of complications, a majority of horses that survived to the time of hospital discharge remained alive at 1 year after surgery (89.7%). No evidence was found regarding the association of variables related to patient signalment, lesion type, surgical procedures, or distance travelled from stable to hospital and long-term survival.

Colic surgery outcomes have been evaluated by numerous previous investigators (6, 7, 10–12, 16, 19–23, 31–34) yet there have been few reports of long-term outcomes after discharge from the hospital for horses undergoing surgery within the past 10 years (7, 10, 17, 21). The most recent report from a population of horses in the US was published approximately 10 years ago, evaluating horses from 2005–2010 and reported survival data after various types of small intestinal anastomoses and resections (21). Given the improvements in surgical techniques and critical care support for horses that have occurred in recent years, the data presented here should contribute important information to help guide horse owner decision-making relevant to colic surgeries for their horses.

The signalment of horses in this study was comparable to the reported US demographic distribution (29) with the most popular breed being the Quarter Horse, at 40.5%, compared to the national average of 42.1%, followed by the American Paint Horse (10.3% vs. national average 7.4%). There was a slightly greater population of Warmblood breeds (7.6%) compared with the national average (3.2%). This likely influenced the distribution of equine athletic activities of the study population, where 25.4% of our population were English style competition horses.

A larger percentage of horses in this study were engaged in competitive equine activities than has been reported as a US average (29) (39.5 and 9.7%, respectively) and none of the horses were reported to be engaged in farm or ranch work. The population of horses used primarily for breeding was slightly higher than the national average at 11.3% compared to 8.5%. Horses used primarily for pleasure riding were represented in numbers consistent with the national average (43.7 and 47.2%, respectively). With the similarities of breed and working populations seen between our population and the national average, it is believed this study may have relevance for many horse populations in the US.

Short-term survival reported for horses in this study, defined as survival to discharge from the hospital for all horses that underwent colic surgery, was 57.8%, which is similar to previously reported rates of 51–62% (10–12, 34–37). When only horses recovering from anesthesia are included in the analysis, short-term survival is reported to be higher at 68–100% (10, 11, 16, 17, 38). The short-term survival including only those horses that recovered from anesthesia was 79.1% in this study. Reporting rates from the time of recovery from anaesthesia removes horses that were euthanized or died during surgery or recovery from the analysis, leading to a favourable selection bias for short-term survival. In the present study, the intraoperative euthanasia rate was 25.9%, which is higher than recent reported rates of 3.8–25.8% (7, 10, 16, 22, 33). The increased intraoperative euthanasia rate reported here may reflect the large distances these horses travelled before surgery. Distance and time of travel from stable to hospital impacts short-term and long-term survival after colic surgery. In one study, travel for more than 70 km (equivalent to 42 miles) was associated with poorer surgical outcomes (33). While travel distance for the horses in this study was not clearly associated with either short-term or long-term outcomes, the majority of horses travelled a long distance to the hospital, (median travel distance of 121 miles, IQR = 93.5–163 miles) exceeding the 42 miles previously associated with better short-term outcome (33).

Of the owners contacted for follow-up information, 67.0% responded, which comparable to other questionnaires for horse owners that were seeking to obtain follow-up information about horses with colic (48.5–92.5%) (6–8, 10, 18). A higher response rate from horse owners would have increased sample size and statistical power. Ideally, long term survival studies should be longitudinal and prospective in design.

When all horses discharged from hospital for which follow-up data was obtained were considered, survival to 1 year after surgery was 89.7% (*n* = 61/68), which is comparable to results in other recently published studies in which survival was measured at the same time point (83.9–96.2%) (4, 6, 7, 10). There were no demographic or surgical variables that were statistically significantly associated with long-term survival in this study, consistent with Immonen’s findings and likely reflective of the overall high number of long-term survivors in both studies (7).

The most frequently reported post-surgical complication reported for horses that have undergone colic surgery is colic (7, 8, 10, 17, 18, 21, 23). A study by Mair and Smith (8) found that 35.1% of horses experienced at least one episode of colic in the first year following surgery and 11% of these horses experienced recurrent colic or severe colic requiring surgery or euthanasia. Other studies reported colic after hospital discharge at variable rates ranging from 20–50% (7, 10, 18, 23). The incidence of post-surgical colic in the present study was 29.6% across the study period (1 month to 7 years) consistent with that reported previously. Many previous reports only considered colic episodes that occurred within the first year after discharge from the hospital. Post-operative colic incidence is reported was previously reported to have an association with higher death rates (8).

Reports of post-surgical colic that occurs after discharge from the hospital are reliant upon the recognition of signs of abdominal pain by the horse’s owner or caretaker. Many of these episodes are mild and a veterinarian is not directly involved in treatment of the horse. Bowden et al. evaluated owners’ knowledge and opinion related to recognition of colic in the horse and identified significant gaps in knowledge (1). Most owners were confident in their ability to recognise colic and would take steps towards evaluating their horse by assessing heart rate, respiratory rate, temperature, and production of feces, yet when asked to describe normal, were often incorrect (1).

The current study had a lower incidence of surgical site infection (8.5%) compared to previously reported rates of 11–42% (7, 8, 17, 23, 39, 40) as well as a lower incidence of abdominal wall hernia (5.8%) compared to 6–17% (4, 6, 7, 17), although this difference may not be meaningful given the relatively low sample size in the current report. In the past, these complications have been associated with a poorer outcome and decreased likelihood of return to sporting activity or athletic use, which was not appreciated in this current study.

Laminitis, while present in only 3 patients in this report (4.3%), was the complication with the highest fatality rate. Of the three patients that develop laminitis, one horse developed clinical signs immediately after colic surgery, was hospitalized for 4 months for treatment of this complication and was euthanized 4 months after discharge. The other two horses developed clinical signs of laminitis after hospital discharge. One of these horses was hospitalized for 5 days for treatment of laminitis and had no further complications after discharge. The other horse required euthanasia 3 months after onset of clinical signs. The rate of post-operative laminitis in this study was higher than previous reported rates of 0.4–1.4% (7, 10, 41). Despite each individual complication rate being low compared to previous reports, when all complications including colic episodes are evaluated, half of all horses discharged had at least one complication (50.7%).

In horses that survive colic surgery, return to athletic activity or other prior function is often a high priority for owners. Prior studies suggest that return to athletic training occurs within 6 months for 68–84% of horses and return to competition ranged from 70–95% (4, 6, 7, 10, 14, 15, 34). Of these horses, 70–91% achieved at least presurgical levels of athleticism. The present study found the return to athletic activity for 76.4% of horses, with 83.0% returning to the same or an improved level. The population in this study looked at both breeding and athletic activity together which is different to other studies that evaluated return to function for athletic performance. There is little information available about the impact of specific rehabilitation protocols on return to function. Rehabilitation data was not collected in this study.

Of all the respondents, 88.7% answered the question regarding satisfaction following surgery, with 100% of respondents reporting they were satisfied or very satisfied (scores of 4 or 5). This is similar to results from previous reports of client satisfaction after colic surgery (76–96.3%) (4, 6, 7) Owner satisfaction is influenced by duration of hospitalization, total cost of surgery and post-operative care, post-operative complications, and return to work. Of these variables, post-operative performance appears to have a significant effect on satisfaction rates (4). When asked whether owners would choose to perform colic surgery on another horse in a similar situation in the future, 75.4% responded yes. However, 21.7% of respondents stated that they were unsure, and that it would depend on the horse and personal situation at that time, due to the significant financial and emotional investment required. There are no reports, however, of the level of satisfaction of all owners whose horses undergo colic surgery but may not survive. Thus, the current reported satisfaction rates are positively biased.

The most important limitations of this study include the limited geographic range of respondents, the retrospective nature of the study, and the small sample size. Retrospective surveys that rely heavily on obtaining follow-up information from owners or that utilize internet-based survey instruments contain a significant risk for selection, recall, confirmation, and response biases (42). The low sample size and lack of response from 33% of horse owners suggests that caution should be used in interpreting results and in comparing results of this study to previous reports. The information obtained, however, is considered important for improving communication with horse owners considering colic surgery for their horses. This information can be used in the future to design a large multi-center prospective study which is needed to better answer questions related to long-term survival and return to athletic function after colic surgery.

## Data availability statement

The raw data supporting the conclusions of this article will be made available by the authors, without undue reservation.

## Ethics statement

The studies involving humans were approved by Institutional Review Board of Washington State University. The studies were conducted in accordance with the local legislation and institutional requirements. The participants provided their written informed consent to participate in this study.

## Author contributions

LM, MS, and DS contributed to conception and design of the study. LM performed data collection and wrote the first draft of the manuscript. LM and DS participated in data analysis. All authors contributed to the article and approved the submitted version.
