# Supplementary material for: Long-term outcome after colic surgery: retrospective study of 106 horses in the USA (2014–2021)
Source: Front Vet Sci. 2023 Oct 4;10:1235198. doi: 10.3389/fvets.2023.1235198 (PMC10582563; doi:10.3389/fvets.2023.1235198)
Supplement: Supplementary file 1 [file Data_Sheet_1.PDF]

WASHINGTON STATE UNIVERSITY

Introduction

Introduction

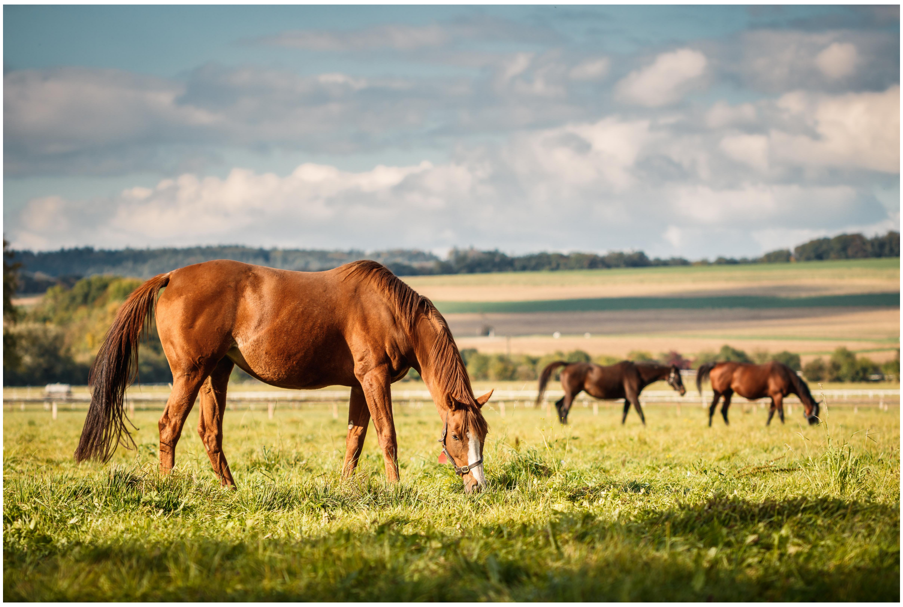

Purpose: Abdominal surgery has been used as a treatment for severe colic episodes in horses for the past 50 years. Understanding the factors that influence survival rates and return to athletic performance after surgery is vitally important in order for veterinarians to provide the best recommendations for treatment of individual horses. There are differences in survival rates and return to athletic function for horses undergoing colic surgery depending on where they live and possibly other, as yet unidentified, factors. Specific data from horses that have undergone surgery at WSU will enable our veterinarians to better advise horse owners in our region as they are making decisions related to colic surgery.

The goals of this work are:

(1) To evaluate short-term and long-term survival rates of horses that have undergone colic surgery at WSU over the past 5 years;
(2) To determine the incidence of complications following colic surgery;
(3) To determine whether horses return to their previous levels of athletic activity after colic surgery; and,
(4) To determine overall level of client (horse owner) satisfaction.

The investigators will use the information derived from this questionnaire to provide more accurate and applicable information to horse owners making decisions regarding colic surgery for their horse at WSU. The collated information from this questionnaire will be published as a scientific, peer-reviewed paper, without identification of any individual horse or horse owner.

Eligibility: Any individual at least 18 years of age who has had a horse that underwent colic surgery at Washington State University between October 1, 2014 and October 1, 2021.

Participation: Participation in this questionnaire is completely voluntary and confidential. We will ask you to provide your name and your horse's name so that we can correlate the information you provide with previously existing information from your horse's medical record at WSU. This information will be treated confidentially. Any public summary of the data, presentation of the data, or publication of the data will contain only anonymized data with no information that might identify you or your horse. You may quit the survey at any time.

Estimated time: This survey takes approximately 10 minutes to complete. There will be questions about the survival time after colic surgery, post-surgical complications, recovery time after surgery, return to athletic activity, and your overall level of satisfaction.

Who: The research team for this project includes Dr. Lisbeth Matthews (Rotating Intern and Masters student at Washington State University), Dr. Debra Sellon (Professor of Equine Medicine at Washington State University), and Dr. Macarena Sanz (Associate Professor of Equine Medicine at Washington State University).

Informed consent: This questionnaire is being done for research purposes. Due to the nature of the survey, questions

regarding possible euthanasia or death of your horse are included and might cause emotional discomfort.

Ethics: This study was deemed exempt from review by the Institutional Review Board (IRB) at Washington State University. This group may be contacted via email at irb@wsu.edu.

If you have any questions, comments, or concerns, please contact Dr. Debra Sellon at dsellon@wsu.edu.

Are you over the age of 18?

- ◯ Yes
- ◯ No

Do you currently reside in the United States?

- ◯ Yes
- ◯ No

Medical Records Identification

In the first section of this survey, we are seeking information that will allow us to match your responses to the medical record of the appropriate horse at the Veterinary Teaching Hospital.

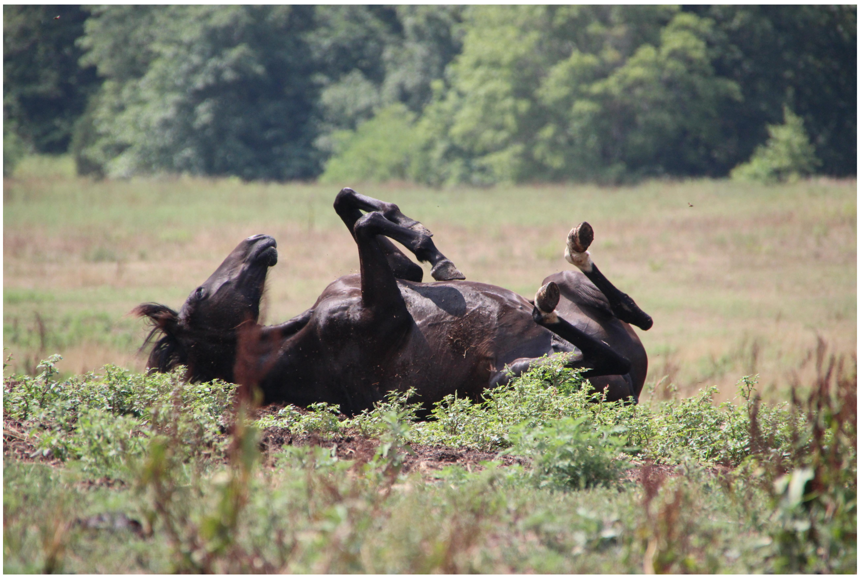

Please confirm your email address.

What is your name?

What is the name of the horse for which you will be providing information (a horse which had colic surgery at WSU in the past 5 years)?

What is your relationship with this horse?

- Owner
- Trainer or legal agent, not owner
- Other, please explain

Post-surgical Complications

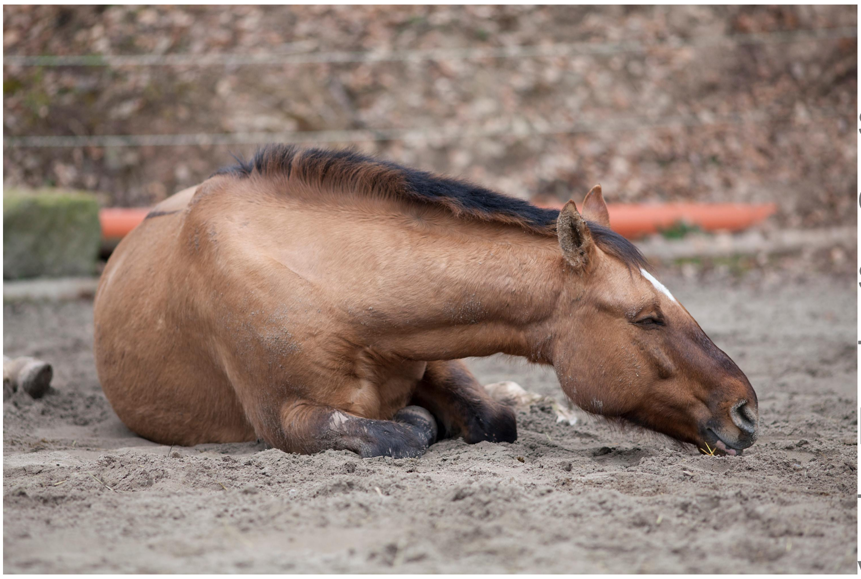

In this section of the survey, we will ask questions about post-surgical complications that occurred after your horse was discharged from the WSU Veterinary Teaching Hospital.

Describe any complications your horse experienced after surgery and discharge from the WSU Veterinary Teaching Hospital. Examples include infection or discharge from the incision, an abdominal wall hernia, laminitis (founder),

infection or swelling at the site of the intravenous catheter, etc. If no complications occurred, please state "no complications".

How many episodes of colic (abdominal pain) has this horse experienced between the date of discharge from the hospital after surgery and the date of this survey?

- 0
- 1
- 2
- 3
- 4+

Consider the most severe episode of abdominal pain or colic which this horse experienced after surgery. On a scale of 0 (no pain) to 10 (worst possible pain), how painful was this horse at that time?

0 1 2 3 4 5 6 7 8 9 10

Most severe episode of colic - degree of pain

Has your horse undergone another abdominal surgery for colic since discharge from the Veterinary Teaching Hospital for the first colic surgery?

- Yes, if so please specify approximate date
- No

Return to Physical Activity

In this section of the survey we will ask questions about your horse's return to physical activity after surgery.

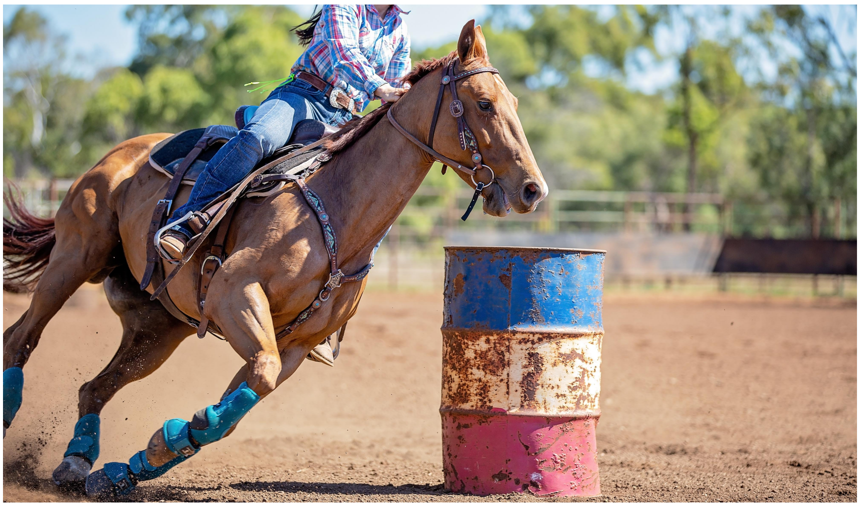

Did your horse return to any type of athletic activity at any time after surgery?

- Yes
- No

◯ Not yet, but it is still a possibility

You indicated that your horse never returned to any type of athletic activity after surgery. What is the reason for this lack of athletic activity?

◯ This horse was unable to return to physical activity because of complications related to the surgery. Please explain.

[ ]

◯ This horse did not return to physical activity for reasons unrelated to the surgery or any complications of surgery. Please explain.

[ ]

At what time after surgery did your horse return to work?

◯ 3 months
◯ 3 to 6 months
◯ 6 months to 1 year
◯ More than 1 year
◯ [ ] Other, please explain

Compared to pre-surgical activities, which of the following best describes the highest level of athletic activity of this horse after surgery?

- ◯ No change; my horse functions at the same level of athletic activity as prior to surgery
- ◯ Less active or less strenuous than before surgery
- ◯ More active or more strenuous than before surgery

What is the reason for decreased level of athletic activity with this horse since surgery? Click all that apply.

- ☐ Horse was unable to compete athletically at the same level after surgery because of complications or as a direct result of the surgery
- ☐ I was reluctant or unwilling to ask my horse to compete at the same level after surgery, even though the horse seemed physically sound
- ☐ I voluntarily retired my horse because of his/her age
- ☐ I decided I no longer wished to engage in this type or level of equine activity for reasons unrelated to the surgery or the horse's condition
- ☐ [ ] Other, please explain

Describe the most strenuous level of athletic activity of this horse ***after*** surgery. Include a brief description of typical training, competition level and type, other athletic events or activities (ranch work, trail riding, pleasure), etc.

Describe the athletic activity of this horse in the weeks ***prior to*** surgery. Include a brief description of typical training, competition level and type, other athletic events or activities (ranch work, trail riding, pleasure), etc.

Is this horse still alive?

- ◯ Yes
- ◯ No
- ◯ I do not know. Please explain.

How long after surgery did this horse die and what was the probable cause of death? Please indicate whether this horse survived for at least one year after surgery.

Satisfaction

In this final section of the survey we will ask you about your overall level of satisfaction with your decision to have colic surgery on your horse.

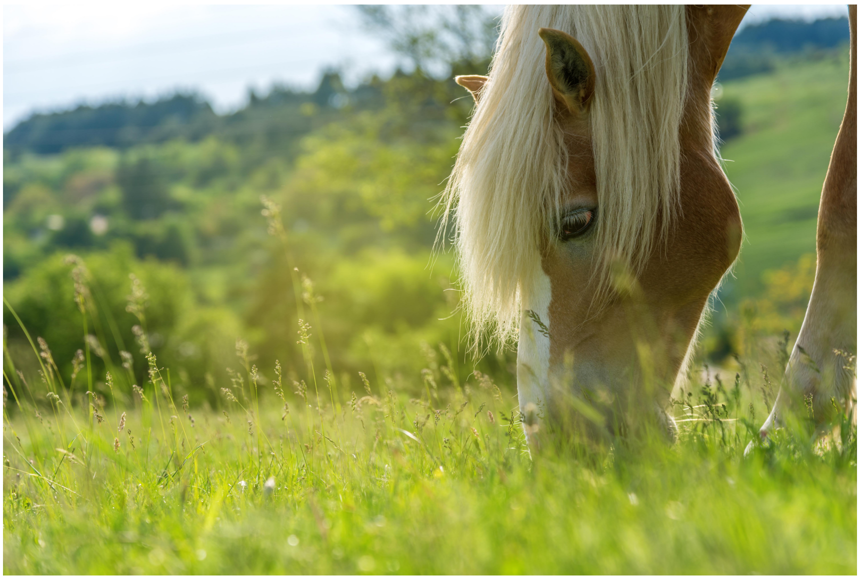

Overall, how satisfied were you with your decision to have colic surgery on this horse?

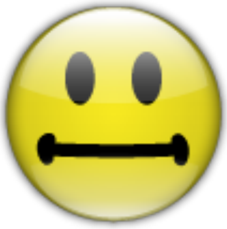

○

In a similar situation in the future, would you proceed with colic surgery for another horse or would you elect euthanasia?

- ○ Yes, I would proceed with surgery
- ○ No, I would not proceed with surgery, I would elect euthanasia
- ○ Unsure

What additional comments would you like to provide regarding your experience with colic surgery with this horse at WSU?

Powered by Qualtrics
